# Peritoneal Dialysis Vintage and Glucose Exposure but Not Peritonitis Episodes Drive Peritoneal Membrane Transformation During the First Years of PD

**DOI:** 10.3389/fphys.2019.00356

**Published:** 2019-04-02

**Authors:** Maria Bartosova, Betti Schaefer, Karel Vondrak, Peter Sallay, Christina Taylan, Rimante Cerkauskiene, Maria Dzierzega, Gordana Milosevski-Lomic, Rainer Büscher, Ariane Zaloszyc, Philipp Romero, Felix Lasitschka, Bradley A. Warady, Franz Schaefer, Akos Ujszaszi, Claus Peter Schmitt

**Affiliations:** ^1^Center for Pediatric and Adolescent Medicine, University Hospital Heidelberg, Heidelberg, Germany; ^2^Department of Pediatrics, Motol University Hospital, Prague, Czechia; ^3^1st Department of Pediatrics, Semmelweis University, Budapest, Hungary; ^4^Pediatric Nephrology, Children’s and Adolescent’s Hospital, University Hospital of Cologne, Cologne, Germany; ^5^Children’s Clinic, Faculty of Medicine, Vilnius University, Vilnius, Lithuania; ^6^Department of Pediatric Emergency, Medicine University Hospital, Polish-American Institute of Pediatrics, Jagiellonian University Medical College, Krakow, Poland; ^7^Nephrology Department, University Children’s Hospital, Belgrade, Serbia; ^8^Pediatric Nephrology, University Children’s Hospital, Essen, Germany; ^9^Department of Pediatrics 1, Strasbourg University Hospital, Strasbourg, France; ^10^Division of Pediatric Surgery, Department of General, Visceral and Transplantation Surgery, University Hospital Heidelberg, Heidelberg, Germany; ^11^Department of General Pathology, Institute of Pathology, University Hospital Heidelberg, Heidelberg, Germany; ^12^Children’s Mercy Kansas City, Kansas City, MO, United States; ^13^Division of Nephrology, University Hospital Heidelberg, Heidelberg, Germany

**Keywords:** peritoneal dialysis, peritonitis, peritoneal membrane, glucose, glucose degradation products, TGF-ß, VEGF, EMT

## Abstract

The impact of peritoneal dialysis (PD) associated peritonitis on peritoneal membrane integrity is incompletely understood. Children are particularly suited to address this question, since they are largely devoid of preexisting tissue damage and life-style related alterations. Within the International Peritoneal Biobank, 85 standardized parietal peritoneal tissue samples were obtained from 82 children on neutral pH PD fluids with low glucose degradation product (GDP) content. 37 patients had a history of peritonitis and 16 of the 37 had two or more episodes. Time interval between tissue sampling and the last peritonitis episode was 9 (4, 36) weeks. Tissue specimen underwent digital imaging and molecular analyses. Patients with and without peritonitis were on PD for 21.0 (12.0, 36.0) and 12.8 (7.3, 27.0) months (*p* = 0.053), respectively. They did not differ in anthropometric or histomorphometric parameters [mesothelial coverage, submesothelial fibrosis, blood, and lymphatic vascularization, leukocyte, macrophage and activated fibroblast counts, epithelial-mesenchymal transition (EMT), podoplanin positivity and vasculopathy]. VEGF and TGF-ß induced pSMAD abundance were similar. Similar findings were also obtained after matching for age and PD vintage and a subgroup analysis according to time since last peritonitis (<3, <6, >6 months). In patients with more than 24 months of PD vintage, submesothelial thickness, vessel number per mmm section length and ASMA fibroblast positivity were higher in patients with peritonitis history; only the difference in ASMA positivity persisted in multivariable analyses. While PD duration and EMT were independently associated with submesothelial thickness, and glucose exposure and EMT with peritoneal vessel density in the combined groups, submesothelial thickness was independently associated with EMT in the peritonitis free patients, and with duration of PD in patients with previous peritonitis. This detailed analysis of the peritoneal membrane in pediatric patients on PD with neutral pH, low GDP fluids, does not support the notion of a consistent long-term impact of peritonitis episodes on peritoneal membrane ultrastructure, on inflammatory and fibrotic cell activity and EMT. Peritoneal alterations are mainly driven by PD duration, dialytic glucose exposure, and associated EMT.

## Introduction

Peritoneal dialysis (PD) provides a cost effective renal replacement therapy independent of a vascular access, greater individual freedom and at least equal patient outcome within the first years of treatment as compared to hemodialysis (van de Luijtgaarden et al., 2016). It is the preferred dialysis modality in young children and is increasingly applied in adults (Mehrotra et al., 2016). PD fluids, however, expose the peritoneal membrane to glucose concentrations 10–50 fold above physiological concentrations and depending on the manufacturing process, to high amounts of toxic glucose degradation products (GDP), to lactate and an acidic pH in single chamber PD solutions. With extended exposure, the peritoneum undergoes profound transformation, including progressive mesothelial cell loss, submesothelial fibrosis, angiogenesis, and vasculopathy. At the time of PD failure, submesothelial blood and lymphatic vessel number is increased (Williams et al., 2002; Braun et al., 2011). These morphological changes result in a gradual increase in small solute transport rates and loss of ultrafiltration (UF) capacity, requiring exposure to an additional glucose load, which within a vicious circle ultimately results in UF and PD failure (Davies et al., 1998, 2001).

Separation of the glucose from the buffer compound, lactate or bicarbonate, allowed introduction of neutral pH, low GDP fluids and raised hope to prevent long term deterioration of the peritoneal membrane, based on numerous *in vitro* and experimental *in vivo* studies (Mortier et al., 2003, 2004, 2005; Grossin et al., 2006; Rippe, 2009; Bajo et al., 2011). Clinical trials revealed higher CA125 effluent concentrations (Haas et al., 2003; Szeto et al., 2007), a putative marker of mesothelial cell viability, and lower hyaluronic acid and procollagen peptide concentrations, suggesting improved peritoneal membrane integrity (Williams et al., 2004). A recent analysis of the peritoneal membrane in children at the time of PD catheter insertion, as well as while on chronic PD with neutral pH, low GDP PD fluids, however, revealed doubling of peritoneal microvascularisation and endothelial exchange area within a few months of PD initiation. These findings independently predicted small solute transport rates. Submesothelial fibrosis progressed less fast (Schaefer et al., 2018). These changes were accompanied by induction of VEGF and TGF-β induced SMAD phosphorylation, by epithelial-mesenchymal transition (EMT), and by inflammatory cell invasion (Schaefer et al., 2018). Lymphatic vessel density and podoplanin positivity – markers of emerging encapsulating peritoneal sclerosis (Braun et al., 2011) – remained largely unchanged. These findings raised questions regarding the assumption of significantly improved biocompatibility as reflected by better preservation of peritoneal membrane integrity and transport function, with neutral pH, low GDP fluids (Blake, 2018). They also emphasized the need for an in-depth analysis of the underlying pathomechanisms, with an ultimate goal of improving PD efficacy and sustainability.

Peritonitis episodes remain a common complication of PD (Andreoli et al., 1999; Mehrotra et al., 2016) and have independently been associated with poorer technique and patient survival (Davies et al., 1996; Boudville et al., 2012; Ye et al., 2017). In an early study comprising 233 PD patients on acidic, high GDP fluids, single peritonitis episodes had no effect on peritoneal small solute transport and UF, whereas recurrent peritonitis episodes increased the D/P creatinine and reduced the UF capacity. Functional changes correlated with the severity of infection as assessed by the cumulative dialysate leukocyte count (Davies et al., 1996). A more recent study with the same fluid type and close monitoring of transport function after the first peritonitis episode suggested recovery of peritoneal small solute transport within 2 weeks post peritonitis, but only subtotal recovery of UF (Ates et al., 2000). In a cohort of 137 patients treated with both low and high GDP fluids and 92 patients with a history of a single episode of peritonitis, the latter exhibited significantly and persistently increased small solute transport, and decreased large molecule transport and UF rates (van Diepen et al., 2015); subgroup analyses with regard to the PD fluid type were not performed.

Data on the impact of peritonitis on peritoneal membrane ultrastructure are few. In Di Paolo et al. (1986) demonstrated peritonitis associated degeneration of the mesothelium and alterations of the connective tissue, which partly resolved within several months of the peritonitis episode. In rodents, acute peritonitis induced loss of mesothelial cells, EMT, and fibrosis (Katz et al., 2012; Balogh et al., 2015). Peritoneal overexpression of the inflammatory cytokines interleukin 1-beta and TNF-alpha, which are upregulated earliest in patients with acute peritonitis (Zemel et al., 1994), induced VEGF and angiogenesis, increased solute permeability and reduced UF (Margetts et al., 2002). We now provide a detailed analysis of the impact of peritonitis episodes on peritoneal membrane integrity, cellular infiltration, EMT, and key cytokine abundance in children treated with neutral pH, low GDP peritoneal dialysis solutions. Children are largely devoid of preexisting comorbidities such as age and life-style related tissue and vascular alterations, mainly suffer from congenital disorders mostly limited to the kidneys and urinary tract (Harambat et al., 2012) and therefore are particularly positioned to facilitate the study of PD treatment and peritonitis induced changes of the peritoneum.

## Materials and Methods

### Biopsy Sampling

Parietal peritoneal biopsy samples from 25 centers were obtained within the scope of the International Pediatric Peritoneal Biobank study. Sampling followed a standardized protocol as described previously (Schaefer et al., 2016). All samples were fixed with needles on cork and instantaneously stored in formalin. After transfer to Heidelberg, parietal tissue samples were embedded in paraffin and underwent immunostaining and digital imaging analysis. Case report forms were provided online via the International Peritoneal Dialysis Network^1^ or by mail and entered into the central data base. The protocol was approved by local ethical boards and performed in accordance with the local national Medical Association’s professional code of conduct (Landesärztekammer Baden-Württemberg) and the declaration of Helsinki. Oral and written consent was obtained from parents and patients as appropriate. The study was registered at www.clinicaltrials.gov (NCT01893710). Between February 2011 and January 2018, 87 parietal peritoneal samples were obtained from 84 children on neutral pH, low GDP PD fluid (45 with pH 7.4 bicarbonate fluid, 15 with pH 7.0 lactate fluid, 24 with pH 7.4 lactate/bicarbonate fluid). The effect of the low GDP PD treatment on peritoneal membrane integrity and function has been published previously (Schaefer et al., 2018). This analysis focused on the distinct effects of peritonitis episodes. Three patients underwent a repeat biopsy, 2 of whom had experienced a single episode of peritonitis before the first sampling. These biopsies were included in the present analysis. The median patient age was 5.7 (2.7, 13.5) years, and PD vintage was 16.9 (7.3, 36.0) months. Daily dialytic glucose exposure was calculated from the most recent, stable PD regime. Two patients with candida peritonitis were excluded from the analysis.

### Immunostaining

All specimens underwent hematoxylin-eosin (H&E) and acid fuchsin orange (AFOG) staining according to standard protocols. Immunostaining was performed with the following antibodies: calretinine, podoplanin (D2-40), CD31, CD45, CD68, ASMA (alpha smooth muscle actin), FSP-1, and cytokeratin. All slides were scanned using Nanozoomer Digital pathology system and analyzed by digital microscopy using Aperio Image Scope version 11. Mesothelial cells integrity was assessed on H&E, calretinine, and podoplanin stained slides and quantified in a semi-quantitative way (0 = no cells present, 1 = 1–24% of the surface covered, 2 = 25–49% of the surface covered, 3 = 50% of cells present, 4 = 51–75%, 5 = 76–95%, 6 = complete coverage). Submesothelial thickness was measured on H&E and CD31 stained slides on at least five different points. Vessels were stained by CD31, lymphatic vessels by D2-40. Quantification was performed by Microvessel algorithm (Aperio Precision Image Analysis), to calculate the number of blood vessels; podoplanin positive lymphatics were subtracted from the CD31 positive vessels (stains both, blood, and lymphatic vessels). Submesothelial microvessel number/mm was calculated as absolute number of vessels in the submesothelial area per 1 mm length of surface peritoneum. Vasculopathy was quantitated as described previously (Honda et al., 2008; Schaefer et al., 2018). Endothelial surface area relative to peritoneal volume was calculated for the total vessel density, blood vessel density, and lymphatic vessel density in a following way: the endoluminal perimeter of CD31/podoplanin stained endothelium ^∗^section thickness ^∗^number of vessels divided by the analyzed peritoneal area ^∗^section thickness (μm^2^/μm^3^). ASMA positivity and CD45 lymphocyte and CD68 macrophage infiltration was quantified in a semi-quantitative way (0–3 score). AFOG stained slides were evaluated for fibrin deposits. EMT cells were determined as previously described using calretinin staining (Yanez-Mo et al., 2003) and findings reconfirmed by cytokeratin and FSP1 co-staining.

### Statistics

Data distribution was assessed graphically and by Shapiro–Wilk test. Data are presented as median with interquartile range (IQR). To assess the differences between the groups, ANOVA or Kruskal–Wallis test were used based on the data distribution. Because of the low sample size, matching by the coarsened exact matching method (Blackwell et al., 2009) was performed for age, PD duration and glucose exposure. The exposure variable was history of peritonitis. Patients without peritonitis were matched to patients with any positive number of previous peritonitis episode. Multivariable linear regression analyses in a forward entry, starting with a univariate analyses were performed to test the PD characteristics with the peritoneal morphology in patients with and without peritonitis; log transformation was performed in case of unequal data distribution. Analyses were performed by STATA13 software (StataCorp, College Station, TX, United States), two-sided tests were performed.

## Results

### Patient Population

Out of 82 patients included in the analysis, 37 patients had a history of peritonitis, and 16 out of the 37 had two or more peritonitis episodes. Time interval between the last peritonitis episode and tissue sampling was 9 (4, 36) weeks. The organisms causing peritonitis were *Staphylococcus* (*aureus*, *warneri*), *Enterococcus faecalis*, *Brevundimonas vesicularis*, *Enterobacter* (*cloacae*, *asburiae*), *Haemophilus influenzae*, *Klebsiella pneumoniae*, *Escherichia coli*, *Streptococcus pyogenes*, and *Pseudomonas* (*stutzeri* and *aeruginosa*). In seven episodes the dialysate culture remained negative and in three episodes the organism was not reported. Patient and PD treatment characteristics are given in Table 1. At the time of biopsy, the PD vintage was 8 months shorter in the peritonitis free patients, while dialytic glucose exposure and anthropometric parameters were similar in both groups.

**Table 1:** Patient and PD treatment characteristics and blood biochemistry at time of biopsy.

	No peritonitis	Previous peritonitis	*p*-value
Patients (n)	47	38	
Age (years)	6.2 (2.7, 11.8)	5.3 (2.4, 13.5)	0.87
Gender (% female)	48%	44%	0.75
BSA (m^2^)	0.8 (0.5, 1.1)	0.8 (0.5, 1.4)	0.57
PD duration (months)	12.8 (7.3, 27.0)	21.0 (12.0, 36.0)	0.053
Glucose exposure (g/m^2^/day)	100 (91, 160)	105 (95, 127)	0.75
Urine output (ml/24 h)	800 (213, 1225)	500 (150, 1100)	0.51
Anuric (%)	19	39	0.064
Creatinine (mg/dl)	8 (4.2, 11.27)	5.05 (3.34, 8.61)	0.22
Albumin (g/l)	37 (34, 40)	35 (31, 41)	0.51
BUN (mg/dl)	57 (38, 67)	42 (21, 57)	0.09
Hemoglobin (g/dl)	10.2 (9.4, 11.5)	10.8 (9.5, 11.7)	0.66
Calcium (mmol/l)	2.4 (2.35, 2.5)	2.4 (2.3, 2.5)	0.33
Phosphorus (mmol/l)	1.73 (1.44, 2.05)	1.65 (1.38, 1.93)	0.53
PTH (pmol/l)	18.3 (12.5, 42.9)	21.4 (10.3, 40.5)	0.75
Bicarbonate (mmol/l)	24 (21, 26)	23 (21, 27)	0.96


*BSA, Body surface area; BUN, blood urea nitrogen; PTH, Parathyroid hormone.*

### Histological Findings

Despite a longer PD duration, patients with a history of peritonitis did not differ in any of the histomorphometric parameters from peritonitis free patients (Table 2). The extent of mesothelial cell loss, submesothelial fibrosis and hypervascularization (i.e., blood and lymphatic vessel density per mm^2^ peritoneal surface area and per mm submesothelial tissue section length) were comparable, as were the respective endothelial surface areas available for peritoneal fluid and solute transport. Both patients groups had a similar lumen over vessel ratio. This L/V ratio was lower as previously reported for children with normal renal function and children with CKD5 (Schaefer et al., 2018), i.e., the PD patients exhibited significant peritoneal vasculopathy. The relative proportion of patients with ASMA positive, activated submesothelial fibroblasts, CD45 positive leukocytes, and CD68 positive macrophages were comparable, the differences in respective semiquantitative cell scores did not reach statistical significance in unadjusted and multivariable adjusted models (Table 2 and Supplementary Table S1). EMT cells, key cells in the peritoneal transformation process, and key cytokines associated with peritoneal angiogenesis, VEGF, and with fibrosis, TGF-ß induced pSMAD, were not different in the two groups.

**Table 2:** Characteristics and morphological findings of the patients treated with low GDP PD with and without history of peritonitis.

	No peritonitis	Previous peritonitis	*p*-value
Tissue samples (n)	47	38	
Mesothel absent	42%	43%	0.87
Mesothel score (0–6)	1.5 (0, 3.5)	1 (0, 3)	0.33
Submesothelial thickness (μm)	352 (264, 475)	419 (264, 550)	0.49
Microvessel density (/mm^2^)	184 (112, 325)	169 (102, 251)	0.50
Submesothelial microvessel number (/mm)	60 (33, 138)	80 (30, 115)	0.60
Lymphatic vessel density (/mm^2^)	24 (12, 48)	29 (21, 43)	0.49
Diffuse podoplanin staining	25%	26%	0.94
Blood cap. vessel density (/mm^2^)	176 (74, 270)	143 (66, 266)	0.52
Total endothelial surface area (μm^2^/μm^3^)	10.0 (7.0, 18.3)	9.6 (5.7, 13.3)	0.35
Lym. endothelial surface area (μm^2^/μm^3^)	1.9 (0.9, 4.2)	2.0 (1.3, 3.6)	0.75
Blood cap. end. surface area (μm^2^/μm^3^)	8.1 (5.4, 12.9)	6.8 (2.1, 13.0)	0.25
L/V ratio	0.4 (0.2, 0.5)	0.4 (0.3, 0.5)	0.76
ASMA positivity (%)	47%	59%	0.27
ASMA score (0–3)	0 (0, 1)	1 (0, 2)	0.081
CD45 positivity (%)	66%	65%	0.97
CD45 score (0–3)	1 (0, 2)	1 (0, 2)	0.075
CD68 positivity (%)	60%	70%	0.18
CD68 score (0–3)	1 (0, 1.5)	1 (0, 2)	0.075
Fibrine positivity (%)	21%	22%	0.93
EMT presence	33%	41%	0.49
EMT (cells/mm^2^)^∗^	30.0 (15.0, 79.6)	18.0 (5.0, 60.3)	0.22
VEGF (% submesothelial area)	33.6 (17.6, 57.0)	32.4 (20.3, 42.9)	0.35
pSMAD (% submesothelial area)	14.2 (5.4, 27.0)	20.6 (8.7, 28.6)	0.19


*Cap., capillary; end., endothelial; submes., submesothelial; lym., lymphatic; L/V ratio, lumen diameter/vessel ratio; ASMA, alpha smooth muscle actin; EMT, epithelial to mesenchymal transition. VEGF, vascular endothelial growth factor. ^∗^Only cell scores from EMT positive patients included.*

After matching groups for age, PD duration and PD related daily glucose exposure and age, all 23 histomorphometric, cellular, and inflammatory parameters were comparable (Table 3).

**Table 3:** Comparisons of age, PD-vintage and dialytic glucose exposure matched low GDP PD patients with and without history of peritonitis.

	No peritonitis	Previous peritonitis	*p*-value
Patients (n)	24	24	
Age (years)	4.0 (1.8, 9.4)	3.3 (1.5, 10.1)	0.71
Gender (% female)	46%	58%	0.39
BSA (m^2^)	0.6 (0.4, 1.2)	0.6 (0.5, 1.0)	0.88
Urine output (ml/24 h)	1050 (400, 1425)	500 (300, 1100)	0.45
Anuric (%)	29	47	0.27
PD duration (months)^∗^	11.3 (8.5, 21.4)	12.0 (8.5, 22.4)	0.66
Glucose exposure (g/m^2^/day)	97 (89, 132)	100 (85, 108)	0.64
Mesothel absent	46%	38%	0.53
Mesothel score (0–6)	0.5 (0.0, 3.5)	1.0 (0.0, 3.0)	0.91
Submesothelial thickness (μm)	304 (200, 358)	413 (250, 500)	0.24
Microvessel density (/mm^2^)	200 (107, 325)	170 (97, 318)	0.82
Submesothelial microvessel number (/mm)	59 (32, 75)	82 (30, 116)	0.21
Lymphatic vessel density (/mm^2^)	39 (23, 56)	33 (22, 46)	0.41
Diffuse podoplanin staining	33%	23%	0.42
Blood cap. vessel density (/mm^2^)	176 (71, 328)	139 (66, 362)	0.72
Total endothelial surface area (μm^2^/μm^3^)	10.0 (7.7, 19.0)	10.2 (5.9, 16.4)	0.82
Lym. endothelial surface area (μm^2^/μm^3^)	3.4 (1.8, 5.7)	2.6 (1.3, 4.4)	0.30
Blood cap. end. surface area (μm^2^/μm^3^)	8.0 (4.1, 12.8)	6.7 (3.3, 15.7)	0.89
L/V ratio	0.4 (0.2, 0.5)	0.4 (0.3, 0.5)	0.28
ASMA positivity (%)	54%	58%	0.77
ASMA score (0–3)	1 (0, 1)	1 (0, 2)	0.55
CD45 positivity (%)	83%	71%	0.30
CD45 score (0–3)	1 (1, 1.5)	1 (0, 2)	0.89
CD68 positivity (%)	67%	79%	0.33
CD68 score (0–3)	1 (0, 1.5)	2 (1, 2)	0.11
Fibrine positivity (%)	25%	25%	1.00
EMT presence	46%	42%	0.77
EMT (cells/mm^2^)^∗∗^	49 (20, 198)	21 (8, 65)	0.34
VEGF (% submes. area)	32.2 (19.2, 63.1)	35.0 (20.3, 51.1)	0.50
pSMAD (% submes. area)	18.1 (6.2, 29.1)	20.3 (7.3, 26.7)	0.65


*Cap., capillary; end., endothelial; submes., submesothelial; lym., lymphatic; L/V ratio, lumen diameter/vessel ratio; ASMA, alpha smooth muscle actin; EMT, epithelial to mesenchymal transition. VEGF, vascular endothelial growth factor. ^∗^At time of biopsy. ^∗∗^Only cell scores from EMT positive patients included.*

To account for potential temporal differences, a subgroup analysis was performed according to the time since last peritonitis (<3, <6, >6 months), and again, all findings were comparable in both groups.

When stratifying patients according to the number of peritonitis episodes experienced, the mesothelium was significantly less well preserved in patients with two and more peritonitis episodes and the L/V ratio (e.g., vasculopathy) was more severe (Table 4). These patients, however, were on PD for a longer period of time and the difference did not persist in multivariable analysis when PD duration was included as an independent variable (Supplementary Table S2b).

**Table 4:** Comparisons of PD patients according to the number of previous peritonitis episodes.

	No peritonitis	1 peritonitis	≥ 2 peritonitis	*p*-value
Tissue samples (n)	47	22	16	
Age (years)	6.1 (2.6, 11.3)	6.2 (1.6, 16.4)	6.7 (3.1, 13.2)	0.96
Gender (% female)	46%	55%	38%	0.58
BSA (m^2^)	0.8 (0.5, 1.1)	0.9 (0.5, 1.5)	0.8 (0.6, 1.1)	0.74
PD duration (months)^∗^	12.4 (7.3, 24.5)	13.8 (9.0, 26.0)	32.9 (21.0, 70.0)	0.018
Glucose exposure (g/m^2^/day)	99.7 (91.6, 177.6)	101.8 (96.3, 117.1)	111.9 (94.8, 153.8)	0.62
Mesothel absent	39%	27%	71%	0.037
Mesothel score (0–6)	1.5 (0.0, 4.0)	2.0 (0.0, 3.0)	0.0 (0.0, 1.0)	0.045
Submesothelial thickness (μm)	352 (258, 450)	305.3 (267, 500)	510 (300.2, 767.3)	0.21
Microvessel density (/ mm^2^)	194 (115, 336)	159 (95, 211)	192 (150, 271)	0.15
Microvessel number (/mm)	61.3 (32.8, 155,3)	50.4 (27, 82.1)	96.2 (79.9, 179,4)	0.059
Lymphatic vessel density (/mm^2^)	24.4 (12.0, 48.2)	22.7 (18.5, 35.4)	33.3 (28.3, 46.3)	0.44
Diffuse podoplanin staining	22%	30%	29%	0.71
Blood cap. vessel density (/mm^2^)	175.7 (74.1, 269.5)	128.6 (63.2, 190.0)	188.1 (123.9, 406.2)	0.32
Total end. surface area (μm^2^/μm^3^)	10.1 (7.4, 18.7)	9.0 (5.2, 11.8)	10.2 (8.5, 15.5)	0.17
Lymphatic end. surface area (μm^2^/μm^3^)	1.9 (0.9, 4.2)	1.7 (1.2, 3.7)	2.5 (1.6, 2.9)	0.94
Blood cap. end. surface area (μm^2^/μm^3^)	8.1 (5.4, 12.9)	4.6 (2.1, 8.9)	7.7 (4.0, 15.7)	0.20
L/V ratio	0.4 (0.3, 0.5)	0.5 (0.4, 0.5)	0.3 (0.2, 0.4)	0.018
ASMA positivity (%)	48%	59%	53%	0.68
ASMA score (0–3)	0.0 (1.0, 1.0)	1.0 (0.0, 2.0)	1.0 (0.0, 3.0)	0.42
CD45 positivity (%)	67%	68%	65%	0.97
CD45 score (0–3)	1.0 (0.0, 2.0)	1.5 (1.0, 2.0)	1.0 (0.0, 2.0)	0.76
CD68 positivity (%)	57%	77%	65%	0.25
CD68 score (0–3)	1.0 (0.0, 2.0)	1.5 (1.0, 2.0)	1.0 (0.0, 2.0)	0.15
Fibrine positivity (%)	22%	18%	24%	0.91
EMT presence	33%	41%	41%	0.72
EMT (cells/mm^2^)^∗∗^	35.0 (10.0, 95.0)	23.0 (6.1, 45.3)	15.0 (1.0, 60.3)	0.45
VEGF (% submes. area)	33.6 (17.6, 57.0)	33.3 (20.3, 42.2)	28.7 (21.1, 49.5)	0.64
pSMAD (% submes. area)	15.3 (5.8, 27)	13.6 (6.6, 26.2)	25.8 (12.2, 33.6)	0.068


*Cap., capillary; end., endothelial; submes., submesothelial. L/V ratio, lumen diameter/vessel ratio; ASMA, alpha smooth muscle actin; EMT, epithelial to mesenchymal transition. VEGF, vascular endothelial growth factor. ^∗^At time of biopsy. ^∗∗^Only cell scores from EMT positive patients included.*

Patients who have been on PD for at least 24 months and had a history of peritonitis had a higher submesothelial thickness and a higher number of vessels per mm section length, but did not differ in the vessel density per section area compared to patients with a similar dialysis vintage and no history of peritonitis (Table 5). They were also more likely to have activated ASMA positive submesothelial fibroblasts compared to peritonitis free patients. In multivariable analyses, adjusting for PD duration and glucose exposure, the difference in ASMA positivity remained significant (*p* = 0.012).

**Table 5:** Comparisons low GDP PD patients with and without history of peritonitis, who were on PD for at least 24 months.

	No peritonitis	Previous peritonitis	*p*-value
Tissue samples (n)	14	16	
Age (years)	7.4 (4.4, 16.7)	13.2 (6.7, 18.0)	0.35
Gender (% female)	42%	38%	0.82
BSA (m^2^)	0.7 (0.6, 1.3)	1.0 (0.8, 1.3)	0.20
PD duration (months)^∗^	37.6 (30.3, 48.0)	52.0 (32.9, 73.0)	0.30
Glucose exposure (g/m^2^/day)	124.4 (64.3, 216.2)	119.8 (111.9, 146.2)	0.82
Mesothel absent	50%	76%	0.32
Mesothel score (0–6)	0 (0, 2)	0 (0, 1.5)	0.42
Submesothelial thickness (μm)	314 (223.3, 511.0)	527 (308.5, 844.0)	0.058
Microvessel density (/mm^2^)	157.4 (97.9, 278.7)	192.4 (149.7, 236.6)	0.96
Submesothelial microvessel number (/mm)	43.4 (26.0, 66.5)	85.9 (53.1, 179.4)	0.014
Lymphatic vessel density (/mm^2^)	53.5 (14.5, 59.0)	25.1 (14.9, 39.5)	0.41
Diffuse podplanin staining	25%	41%	0.37
Blood cap. vessel density (/mm^2^)	96 (65, 204)	189 (126, 224)	0.57
Total endothelial surface area (μm^2^/μm^3^)	8.2 (5.5, 15.2)	9.7 (8.5, 11.5)	0.93
Lym. endothelial surface area (μm^2^/μm^3^)	4.1 (1.1, 5.8)	1.7 (0.9, 2.5)	0.14
Blood cap. end. surface area (μm^2^/μm^3^)	7.3 (2.3, 9.0)	7.5 (6.8, 10.9)	0.62
L/V ratio	0.5 (0.2, 0.5)	0.4 (0.2, 0.5)	0.52
ASMA positivity (%)	33%	71%	0.017
ASMA score (0–3)	0.0 (0.0, 1.5)	2.0 (0.0, 3.0)	0.56
CD45 positivty (%)	50%	76%	0.23
CD45 score (0–3)	0.5 (0.0, 1.0)	1.0 (1.0, 2.0)	0.79
CD68 positivity (%)	67%	76%	0.73
CD68 score (0–3)	1.0 (0.0, 1.0)	1.0 (1.0, 2.0)	0.14
Fibrine positivity (%)	8%	18%	0.47
EMT presence	25%	53%	0.13
EMT (cells/mm^2^)^∗∗^	20.0 (5.0, 20.0)	11.3 (3.0, 26.6)	0.92
VEGF (% submes. area)	46.6 (16, 49.7)	28.7 (22.2, 42.9)	0.44
pSMAD (% submes. area)	11.7 (9.2, 18.2)	25.8 (8.5, 33.6)	0.11


*Cap., capillary; end., endothelial; lym., lymphatic; submes., submesothelial. L/V ratio, lumen diameter/vessel ratio; ASMA, alpha smooth muscle actin; EMT, epithelial to mesenchymal transition. VEGF, vascular endothelial growth factor. ^∗^At time of biopsy. ^∗∗^Only cell scores from EMT positive patients included.*

In multivariable analyses comprised of data from all biopsies and after adjusting for age, previous peritonitis, dialytic glucose exposure, PD duration and presence of EMT, submesothelial thickness was independently associated with PD duration and the presence of EMT (*p* = 0.002/0.036). Glucose exposure independently predicted peritoneal vessel density and EMT submesothelial microvessel number per mm tissue section (*p* = 0.078 and 0.027) (Supplementary Tables S2a–c). In subgroup analyses, after adjusting for age, glucose exposure, PD duration and EMT, submesothelial thickness was independently associated with EMT in peritonitis free patients (*p* = 0.04), and with duration of PD in patients with previous peritonitis (*p* = 0.01) (Supplementary Tables S2d,e).

## Discussion

This is the first detailed analysis of the long term impact of peritonitis on peritoneal membrane integrity in a substantial number (*n* = 82) of pediatric patients on chronic PD. Patients with and without a history of peritonitis did not differ in any of the histomorphometric parameters, nor in inflammatory cell invasion, EMT, or cytokine expression. These findings suggest that, PD fluid associated toxicity in contrast to peritonitis episodes drives peritoneal membrane transformation.

Experimental *in vivo* studies have previously demonstrated major peritoneal inflammatory and fibrotic changes with bacterial and LPS induced peritonitis (Hautem et al., 2017). Repeated peritoneal equilibration tests suggest rapid recovery of peritoneal solute transport in most patients after a single episode of peritonitis (Davies et al., 1996; Ates et al., 2000), but persistent changes with repeated peritonitis episodes (Davies et al., 1996), ultimately associating with worse technique and patient outcome (Boudville et al., 2012; Ye et al., 2017). Most of these data were obtained in patients treated with acidic, high GDP PD fluids. In a recent RCT, patients treated with neutral pH, low GDP PD fluid experienced less frequent and less severe episodes of peritonitis as compared to patients treated with high GDP fluids (Johnson et al., 2012). Albeit, not analyzed in that trial, the suggestion has been made that the impact of peritonitis episodes on peritoneal membrane integrity and transport function may be less pronounced in patients treated with low GDP fluids. These assumptions, however, could not be reconfirmed in a recent meta-analysis (Cho et al., 2014). Peritoneal biopsies taken from 5 patients treated with high GDP fluids during acute peritonitis and one to 4 months thereafter demonstrated persistent mesothelial alterations and submesothelial sclerotic lesions (Di Paolo et al., 1986).

A more recent study comparing peritoneal morphology of 23 patients on low and 23 patients on high GDP fluid, suggested better preservation of the mesothelial cell layer and less submesothelial fibrosis and vasculopathy with low GDP fluid. These differences, however, were lost when previous peritonitis episodes were taken into account, suggesting that potential benefits of the low GDP fluid are superimposed by untoward peritonitis effects (Del Peso et al., 2016). Our findings in pediatric patients who used low GDP PD fluids do not support the notion of peritonitis related peritoneal sequelae. In parietal peritoneal tissue from patients who had experienced previous peritonitis episodes, we did not observe any differences in histomorphological features, inflammatory cell invasion, in VEGF, and in TGF-ß induced pSMAD abundance and in the degree of EMT, as compared to peritoneal specimens from peritonitis free patients. EMT cells secrete VEGF and TGF-ß (Aroeira et al., 2005, 2007) and thus represent key mediators of the peritoneal transformation process (Yanez-Mo et al., 2003; Lopez-Cabrera, 2014), albeit their origin is debated (Chen et al., 2014). Matching for age to rule out preexisting differences in age related peritoneal thickness and vascularization (Schaefer et al., 2016), as well as for PD vintage and dialytic glucose exposure, reconfirmed the findings. Likewise, analyses according to the time interval since the last peritonitis episode, and the number of peritonitis episodes did not demonstrate any significant differences. In the small subgroup of patients with more than 2 years of PD, peritonitis positive patients exhibited some differences. The likelihood of activated fibroblast positivity persisted in multivariable analysis, suggesting that in patients on long term PD, peritonitis episodes may be associated with some enhanced peritoneal profibrotic activity, but the statistical power of this subgroup analysis is limited. In the multivariable analysis of the entire cohort, peritonitis did not predict any of the key morphological peritoneal parameters.

In contrast to the minor differences attributable to peritonitis history, PD duration and dialytic glucose exposure independently predicted the peritoneal membrane histomorphology. In multivariable analyses of all patients, PD duration predicted submesothelial thickness, and glucose exposure predicted peritoneal vessel density. PD treatment induced EMT (Aroeira et al., 2007) was associated with both submesothelial fibrosis and vessel density. History of peritonitis and the number of previous peritonitis did not predict key histomorphological outcome parameters. In subgroup analyses according to peritonitis history, submesothelial thickening was independently associated with EMT in peritonitis free patients, and with duration of PD in patients with previous peritonitis. The independent association of both submesothelial fibrosis and submesothelial vessel density with the presence of EMT cells points to the key role of EMT in the peritoneal transformation process. EMT, in turn, represents a potential biomarker, and therapeutic target (Aufricht et al., 2017).

Of note, during the past few decades, prevention and treatment of peritonitis has increasingly been guided by evidence based and repeatedly updated international recommendations (KDQQI guidelines) (Li et al., 2016). Not only has the incidence of peritonitis declined (Campbell et al., 2015), but peritonitis episodes are usually recognized early and broad spectrum antibiotic treatment covering most of the bacteria is initiated promptly. None of the centers reported unsuccessful treatment of the peritonitis episodes.

Our study has important limitations. Although this is the largest study on peritonitis induced changes of the peritoneal membrane thus far, the number of patients is not extensive, and thus the sensitivity is low. Moreover, and despite current recommendations (Mujais et al., 2000; National Kidney Foundation, 2006), only a minority of the contributing centers performed PET and therefore the impact of peritonitis episodes on peritoneal membrane function could not be studied comprehensively. On the other hand, strength of the study is the patient population as children are devoid of life style and aging related preexisting tissue damage and the predominant underlying diseases of these patients, such as congenital abnormalities of the kidneys and urinary tract, do not affect peritoneal integrity. This allows for a more sensitive and specific analysis. Whether growing children have a greater plasticity and thus a greater potential to compensate for peritonitis induced transient peritoneal damage is unknown.

In conclusion, our detailed analysis of the peritoneal membrane in pediatric patients on maintenance PD with neutral pH, low GDP fluids, does not support the notion of a consistent long-term impact of peritonitis episodes on the peritoneal membrane ultrastructure, on inflammatory and fibrotic cell activity and EMT. Peritoneal alterations are primarily driven by PD duration and dialytic glucose exposure.

## Data Availability

The datasets generated for this study are available on request to the corresponding author.

## Author Contributions

MB and BS contributed to the conception of the study, collected specimens and patient data, performed immunostainings and digital image analyses, and wrote the manuscript. FL contributed to the histological and digital imaging analyses. KV, PS, CT, RC, MD, GM-L, RB, AZ, PR, and BW collected specimens and clinical data and contributed to the manuscript. BW and FS contributed to the conception of the study. AU contributed to the conception of the study, performed the statistical analysis, and wrote the manuscript. CS conceptualized the study, contributed to specimen sampling, histological and digital imaging analyses and statistical analyses, and wrote the manuscript. All authors approved the final version of the manuscript.

## Conflict of Interest Statement

CS has obtained lecturing honoraria, travel support, and investigator initiated research funding from Fresenius Medical care and lecturing and consulting honoraria from Baxter. The remaining authors declare that the research was conducted in the absence of any commercial or financial relationships that could be construed as a potential conflict of interest.

## Footnotes

1 www.pedpd.org
