# Supplementary material for: Peritoneal Dialysis Vintage and Glucose Exposure but Not Peritonitis Episodes Drive Peritoneal Membrane Transformation During the First Years of PD
Source: Front Physiol. 2019 Apr 2;10:356. doi: 10.3389/fphys.2019.00356 (PMC6455046; doi:10.3389/fphys.2019.00356)
Supplement: Supplementary file 1 [file Data_Sheet_1.PDF]

Supplementary Material

Supplementary Table 1

a) Association of previous peritonitis episode with histomorphological findings. Multivariate linear and logistic regression models are adjusted for age, PD duration and glucose exposure. Reference value is "no previous peritonitis".

	ß-coefficient (unadjusted)	p-value	ß-coefficient (adjusted)	p-value
Log Microvessel density (/mm^2)	-0.15 (-0.45 – 0.15)	0.344	-0.15 (-0.45 – 0.16)	0.340
Log Submesothelial vessel number (/mm)	0.04 (-0.37 – 0.45)	0.848	-0.03 (-0.46 – 0.40)	0.890
Log Blood capillary density (/mm^2)	-0.15 (-0.57 – 0.27)	0.477	-0.11 (-0.55 – 0.31)	0.597
L/V ratio	0.01 (-0.05 – 0.08)	0.672	0.02 (-0.05 – 0.10)	0.499
Submesothelial thickness (µm)	74 (-51 – 202)	0.238	18 (-102 – 137)	0.768
	Odds ratio (unadjusted)	p-value	Odds ratio (adjusted)	p-value
ASMA positivity	1.7 (0.7 – 4.0)	0.250	1.8 (0.7 – 4.5)	0.218
CD45 positivity	0.9 (0.3 – 2.1)	0.756	0.8 (0.3 – 2.0)	0.617
CD68 positivity	1.8 (0.7 – 4.4)	0.229	1.7 (0.5 – 3.4)	0.484
Presence of EMT	1.3 (0.5-3.2)	0.540	1.2 (0.5 – 3.2)	0.672

b) Association of recurrent peritonitis episode with histomorphological findings. Multivariate linear and logistic regression models are adjusted for age, PD duration, glucose exposure. Reference value is "no previous peritonitis". Peritonitis episode stratified to 0, 1, and 2 or more.

	ß-coefficient (unadjusted)	p-value	ß-coefficient (adjusted)	p-value
Log Microvessel density (/mm^2)	-0.04 (-0.23 - 0.15)	0.684	-0.01 (-0.21 - 0.19)	0.912
Log Submesothelial vessel number (/mm)	0.13 (-0.12 - 0.38)	0.299	0.11 (-0.16 - 0.38)	0.427
Log Blood capillary density (/mm^2)	-0.03 (-0.29 - 0.23)	0.809	0.05 (-0.23 - 0.33)	0.713
L/V ratio	-0.02 (-0.06 – 0.03)	0.428	-0.01 (-0.06 – 0.04)	0.708
Submesothelial thickness (µm)	78 (2 - 154)	0.045	30 (-45 - 105)	0.429
	Odds ratio (unadjusted)	p-value	Odds ratio (adjusted)	p-value

ASMA positivity	1.3 (0.7 to 2.2)	0.368	1.3 (0.7 to 2.4)	0.415
CD45 positivity	0.9 (0.5 to 1.6)	0.694	0.8 (0.4 to 1.5)	0.512
CD68 positivity	1.2 (0.7 to 2.2)	0.461	1.0 (0.6 to 2.0)	0.892
Presence of EMT	1.2 (0.7 to 2.1)	0.482	1.1 (0.6 to 2.0)	0.797

Supplementary Table 2

a) Multivariate linear regression analysis of *submesothelial thickness* of all biopsies.

	Coeff.	lower CI 95%	upper CI 95%	p-value
Age (years)	2.88	-6.78	12.56	0.552
Previous peritonitis	35.15	-82.57	152.88	0.553
Dialytic glucose exposure (g/m^2/day)	-0.02	-0.82	0.77	0.950
PD duration (months)	4.32	1.73	6.91	0.001
Presence of EMT	127.25	85.71	392.42	0.041

b) Multivariate linear regression analysis of *total microvessel density* of all biopsies.

	Coeff.	lower CI 95%	upper CI 95%	p-value
Age (years)	-0.017	-0.043	0.009	0.209
Previous peritonitis	-0.151	-0.457	0.155	0.330
Dialytic glucose exposure (g/m^2/day)	-0.001	-0.004	0.000	0.078
PD duration (years)	-0.004	-0.011	0.002	0.235
Presence of EMT	0.089	-0.219	0.399	0.564

c) Multivariate linear regression analysis of *submesothelial microvessel number per mm section length* of all biopsies.

	Coeff.	lower CI 95%	upper CI 95%	p-value
Age (years)	-0.004	-0.039	0.030	0.797
Previous peritonitis	0.032	-0.390	0.455	0.878
Dialytic glucose exposure (g/m^2/day)	-0.002	-0.005	0.001	0.108
PD duration (years)	-0.001	-0.102	0.082	0.820
Presence of EMT	0.493	0.057	0.929	0.027

d) Multivariate linear regression analysis of *submesothelial thickness* in PD patients without a history of peritonitis.

	Coeff.	lower CI 95%	upper CI 95%	p-value
Age (years)	2.8	-9.87	15.49	0.654
Glucose exposure (g/m^2/day)	-0.002	-0.77	0.76	0.995
PD duration (years)	2.47	-1.05	5.98	0.162
Presence of EMT	148.54	6.75	290.33	0.041

e) Multivariate linear regression analysis of *submesothelial thickness* in PD patients with a history of peritonitis

	Coeff.	lower CI 95%	upper CI 95%	p-value
Age (years)	2.39	-13.62	18.39	0.762
Glucose exposure (g/m^2/day)	-0.36	-3.15	2.42	0.790
PD duration (years)	6.26	1.57	10.94	0.011
Presence of EMT	60.39	-172.13	292.93	0.598
